# Phase Behavior of Potential Drug Delivery Systems, Polycaprolactone—Nonsteroidal Anti-Inflammatory Drug in a Pressurized Carbon Dioxide Medium

**DOI:** 10.1021/acsomega.5c06958

**Published:** 2025-12-17

**Authors:** Dóra Arany, Noémi Kurucz, Márton Kőrösi

**Affiliations:** Department of Chemical and Environmental Process Engineering, Faculty of Chemical Technology and Biotechnology, Budapest University of Technology and Economics, Műgyetem rkp. 3., H-1111 Budapest, Hungary

## Abstract

Although high-pressure technologies are widely used, for example, in the polymer and pharmaceutical industries, measuring phase equilibria in high-pressure media is still challenging, especially for multicomponent systems. Although the most reliable methods in solid–liquid­(-gas) equilibrium measurements, also providing additional information, are high-pressure differential scanning calorimetry (DSC) and transitiometry, the most common techniques for detecting solid–liquid phase transitions in pressurized media are visual observation-based methods. In this research, the melting behavior of potential controlled-release active pharmaceutical ingredient (API) polymer systems (ibuprofen–polycaprolactone, ketoprofen–polycaprolactone) in the presence of high-pressure carbon dioxide was investigated. First, the pressure-dependent behavior of pure compounds was investigated by using two different methods. One is based on differential pressure measurement, developed by the research group, and the other is the widely used visual observation-based process. The API polymer systems were investigated in a high-pressure view-cell. Both systems showed eutectic behavior. According to the measurement data, eutectic and melting temperatures of the system were highly dependent on pressure, but the eutectic composition was found to occur at a nearly constant value. The thermodynamic data from the research could be applicable to high-pressure particle formation technologies, such as the particle from gas saturated solutions (PGSS).

## Graphical abstract

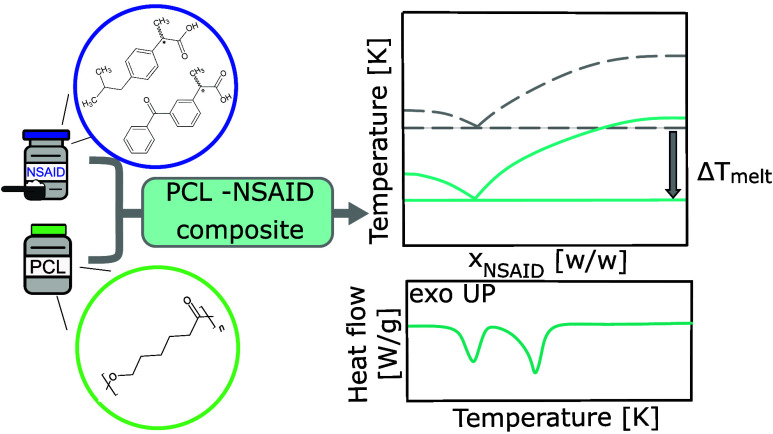

## 1 Introduction

While the thermodynamic foundations of supercritical fluids (those with pressure and temperature both above the compound’s critical values) were laid at the end of the 19th century, their industrial application has also been significant in recent decades.^1^ Their main advantage is that their thermodynamic parameters, such as diffusivity, density, and viscosity, can be controlled near the critical point by adjusting pressure and temperature.^2^

Technologies employing high-pressure fluids aim for special products otherwise not achievable or properties that are not afforded by conventional techniques. They may also reduce energy consumption or, in some cases, even replace organic solvents. Supercritical propane is used in extraction processes in bitumen deasphalting.^3^ Sub- and supercritical water (SCW) is also used in special cases, such as the disposal of hazardous wastewater, conversion of biomass to energy,^4^ or semicontinuous hydrothermal waste valorization.^5,6^

Although carbon dioxide is known as a greenhouse gas, it is the most widely used high-pressure solvent on an industrial scale. The most known technology is the SC–CO_2_ extraction of caffeine from coffee beans,^7^ where carbon dioxide acts as a solvent. It is also highly effective for obtaining specific herbal fractions, such as CBD oil, which has recently gained significant popularity for its numerous beneficial effects.^8,9^ Supercritical CO_2_ has already been industrially used as a solvent in wood impregnation,^10^ but similar processes also enable the homogeneous incorporation of active pharmaceutical ingredients into the internal structure of various implants,^11^ patches,^12,13^ and contact lenses.^14^ The good diffusion properties of high-pressure carbon dioxide make it possible to deliver relatively large amounts of drug to the inside of the carrier. SC–CO_2_ is also used in the polymer industry as a temporary plasticizer^15,16^ and during the foaming of thermolabile plastics (*e.g*., polylactic acid).^17,18^ In addition to the aforementioned applications, controlled particle formation processes using supercritical CO_2_ also have relevance in the pharmaceutical industry. Due to its wide range of applications, carbon dioxide can serve various roles across different technologies.^19,20^ The relevant particle formation technologies and the thermodynamic data needed for each process using dense CO_2_ are summarized in Table 1.

**Table 1:** CO_2_-Assisted Micronization Processes

	Rapid Expansion of Supercritical Solutions (RESS)	Gas and Supercritical Antisolvent Precipitation (GAS, SAS)	Particles from Gas Saturated Solutions (PGSS)
Role of CO_2_	solvent	antisolvent (precipitation agent)	solute
Principle of operation	The solid sample is dissolved in a high-pressure medium, which is sprayed to atmospheric pressure, where the dissolving power of the medium is suddenly drastically reduced, resulting in the formation of a small particle size product.	The solid sample is dissolved in a good solvent, then large oversaturation is achieved by contacting the organic solution with dense CO_2_, thus expanding the solvent and reducing polarity, resulting in the precipitation of a small particle size product.	The process involves the expansion of a carbon dioxide–saturated melt to atmospheric pressure, which allows the creation of particles with a well-controllable size range.
Phase equilibria data needed for design	Solubility data of the compound in CO_2_	Vapor–liquid equilibrium data, solubility data of the compound in CO_2_ pressure-dependent melting temperature data to avoid the precipitation of a melt	Solubility data of CO_2_ in the compound pressure-dependent melting temperature data
References	^21–23^	^24,25^	^26,27^

Considering the applications of high-pressure CO_2_ making use of its plasticizing properties and/or resulting in the formation of solid (even particulate) materials, gathering phase equilibrium information on the expected solid–liquid phase transitions may prove highly useful in setting the operational boundaries of said processes. The melting temperature under high-pressure carbon dioxide is affected by two concurrent phenomena. One is melting point depression, caused by the dissolution of carbon dioxide (or another soluble medium) in the solid sample or its melt. This effect dominates the melting temperature–pressure tendency in the lower pressure range. The concurrent effect of static or mechanical pressure increases the melting temperature of most compounds, based on the Clapeyron equation. Usually, the effect of static pressure dominates the tendency of melting temperature at higher pressures.^28^ Commercially standardized measurement methods in the field, such as high-pressure differential scanning calorimetry (HP-DSC) or transitiometry, yield the most information. Using HP-DSC, the information about thermal events can be extended by their specific enthalpy change beyond their temperature,^29^ while transitiometers allow the detection of volumetric changes, as well.^30^ But, due to their relatively high prices, the challenges of determining melting temperature under pressurized media were responded to by the emergence of numerous measurement techniques, adapting existing apparatuses or creating entirely new methodologies.^31–34^ Solid–liquid–gas equilibrium measurements are most often done in a high-pressure view-cell, which allows visual observation of phase transitions, even under high-pressure conditions. In their simplest form, evaluation is based purely on visual observation by the operator,^28,35^ but there are several modifications, such as the installation of a camera^36^ or a polarized microscope,^37^ increasing accuracy. In addition to standalone studies on melting behavior, the equipment also allows for observations, often complementing measurements in automated devices.^38^ Besides solid–liquid–gas phase equilibria, the view-cell is widely applicable and adaptable to other high-pressure phase equilibrium measurements. Its most widespread application is to measure multicomponent vapor-liquid phase equilibria, by cloud point and redissolution point detection or by sampling equilibrium phases.^39,40^

In addition, special methods are also known, such as torsional braid analysis, where relative rigidity and mechanical damping are detected as a function of temperature.^18^ Another technique is pressure change detection, where the combined effect of CO_2_ dissolution into the solid sample and/or its melt and molar volume change is observed during melting or freezing under high-pressure CO_2_.^41^ The pressure change is almost negligible compared to system pressure and is challenging to detect. Hence, a method based on differential pressure measurement has been developed for sharper detection. The base concept of the method is measuring pressure difference between a reference and a sample holder cell is monitored as a function of temperature.^38^

In this paper, the solid–liquid phase transition of potential drug delivery systems was investigated with two different high-pressure analytical methods. Melting behavior data may be used for designing micronization processes. Two active pharmaceutical ingredients (APIs), ibuprofen and ketoprofen, were chosen, and the same polymer carrier, polycaprolactone (PCL), was applied. Ibuprofen and ketoprofen are both chiral compounds and a common nonsteroidal anti-inflammatory drug (NSAID), administered as racemates. Their schematic molecular structures are shown in Figure 1.

**Figure 1:**
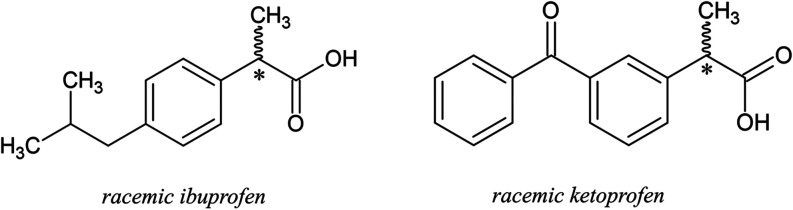
Molecular structures of racemic ibuprofen and ketoprofen.

Ibuprofen is also a well-known model material, the pressure-dependent melting behavior of which has previously been investigated in various research groups.^37,42^ To the best of the authors’ knowledge, in the case of ketoprofen, there is no pressure-dependent melting temperature data available in the literature.

The polymer of choice is polycaprolactone, a low-melting-point inert biopolymer, which is used both in implants and as a drug carrier.^43^ The schematic molecular structure of the compound is shown in Figure 2.

**Figure 2:**
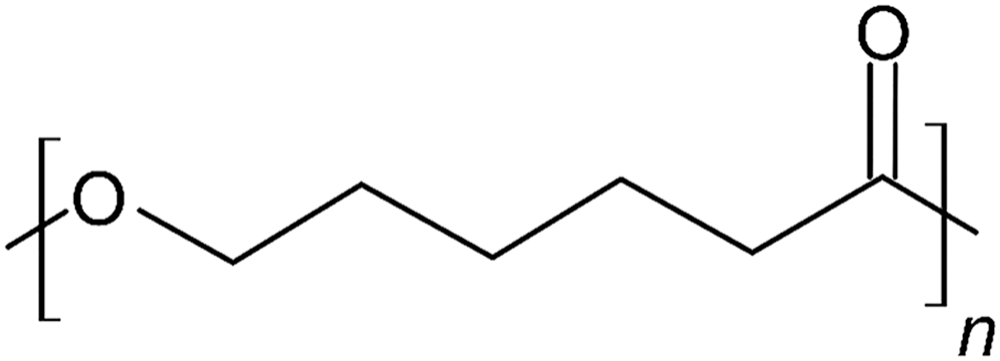
Molecular structure of polycaprolactone.

## 2 Materials and Methods

### 2.1 Materials

Racemic ibuprofen (CAS NR 15687–27–1, purity >98%, GC, Lot I2HJD IQ) and racemic ketoprofen (CAS NR 22071–15–4, purity >98% Lot B5ZHG HG IQ, Tokyo, Japan) were bought from Tokyo Chemical Industry (Tokyo, Japan). Polycaprolactone (CAS NR 24980–41–4, purity >99%, Source MKCS9213) was supplied by Sigma-Aldrich (Darmstadt, Germany).

Chloroform (CAS NR 67–66–3, purity >99.9% (GC) with 0.6–1% ethanol stabilizer) was purchased from Molar Chemical (Halásztelek, Hungary). Carbon dioxide (Biogon C) used in the experiments was bought from Linde Gas Hungary (Répcelak, Hungary) and had a purity of over 99.9%. Material properties are summarized in Table 2.

**Table 2:** Materials

Chemical name	CAS number	Source	LOT number	Purity	Analysis method
Ibuprofen ((*RS*)-2-(4-(2-methylpropyl)phenyl) propanoic acid)	15687–27–1	TCI	I2HJD IQ	>0.98	GC
Ketoprofen (*RS*)-2-(3-benzoylphenyl) propanoic acid)	22071–15–4	TCI	B5ZHG HG IQ	>0.98	HPLC
Poly(ε-caprolactone)	24980–41–4	Sigma-Aldrich	MKCS9213	>0.99	GC < 0.5% water
Chloroform (trichloromethane)	67–66–3	Molar Chemical	GV9763786	>0.999	GC
Carbon dioxide	124–38–9	Linde Gas Hungary	1074321821	>0.999	

### 2.2 Methods

#### 2.2.1 Sample Preparation for the High-Pressure Melting Temperature Measurements

The APIs and the polymer could not be mixed either physically or in a molten state because of the difference in the viscosity of the melts. An organic solvent must be found, which is appropriate for both components. The APIs have several good solvents, but in the case of PCL, chloroform was the best choice.^44^ Pure PCL and ibuprofen or ketoprofen were weighed using an analytical balance in the amount appropriate to the desired composition. The total mass of the samples was approximately 700 mg. The solid sample was then dissolved in 15 mL of chloroform, crystallized, and dried in a Petri dish under ambient conditions.

#### 2.2.2 Visual Observation-Based Measurements in a Variable-Volume View-Cell

Melting temperature measurements were taken using a high-pressure view-cell (New Ways of Analytics GmbH, Lörrach, Germany) with operational parameters of *T*_max_ = 473 K, *p*_max_ = 75 MPa, and an adjustable volume of 45–70 mL. This equipment gives the possibility to observe phase transitions under high-pressure conditions through a sapphire window. Its variable volume (by a piston) allows isobaric heating of the sample and can also be used to modify the volume, thereby adjusting pressure based on experimental requirements.^45,46^ The schematic depiction of the apparatus is presented in Figure 3.

**Figure 3:**
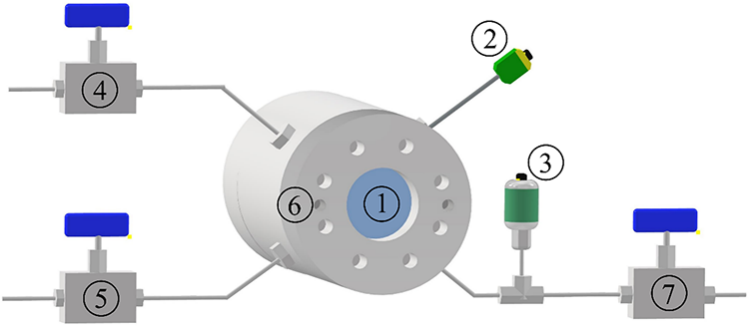
Schematic depiction of the high-pressure view-cell: 1. Sapphire window and piston with window. 2. Temperature transducer. 3. Pressure transducer. 4, 5. Valves. 6. Sockets of the heating rods. 7. CO_*2*_ supply through valve (Teledyne ISCO 260D syringe pump not pictured).

A measurement started with removing the front cover; then, approximately 200 mg of material was measured into a glass sample holder and placed inside the high-pressure view-cell. After the lid was reattached, the system was pressurized through valve number 7 using a Teledyne ISCO 260D syringe pump (Teledyne ISCO, Lincoln, NE, USA, not pictured). After the appropriate pressure was set, the valve was closed, isolating the system from the carbon dioxide supply. After the gas-tightness of the apparatus was ensured, the system temperature was increased by 2 K every 4 min. Pressure, temperature, and volume data were recorded by a computer. In the case of small molecule size, crystalline compounds, two temperatures were recorded for each measurement: the temperature at the first melting point (FMP), which marked the appearance of the first liquid drop, or an observable change related to melting, and finally the temperature at which the whole sample became a liquid, marking the last melting point (LMP). In the case of polymers, measuring the phase transition is a bit more complex: the melting temperature cannot be as sharply defined because of the distribution of the molecule size and its semicrystalline structure (at least in the case of the currently investigated PCL). In the case of polymeric samples, the two recorded temperatures were: the first visible change (the surface of the PCL sample became shiny, and it started to become transparent) and the temperature at which the sample became completely transparent and started to lose its structure. After the measurements, the cell was cooled, depressurized, and cleaned.

The expanded measurement uncertainty for the view-cell method was calculated from the standard deviation with the help of *Student’s t-distribution* table (for α = 0.05). The number of repetitions, the standard deviation, and the expanded measurement uncertainty are shown in Table 3. The calibration of the temperature sensor of the view-cell was verified based on the boiling temperature of multiple pure solvents and the melting temperature of ice. Also, the critical pressure and temperature of carbon dioxide were measured, while the temperatures of the two discussed apparatuses were compared using the measurement data on small-molecule organic samples.

**Table 3:** Measurement Uncertainty of the High-Pressure View-Cell Method

	Ibuprofen	Polycaprolactone	
Pressure	12 MPa (first change)	10 MPa (first change)	10 MPa (melt)
Number of repetitions	3	3	3
Standard deviation	0.25 K	0.31 K	0.64 K
Expanded measurement uncertainty	±1.08 K	±1.31 K	±2.76 K

#### 2.2.3 Measurements Based on Differential Pressure Detection

An automated measurement device was developed in our research group, the schematic depiction of which (from a previous publication^35^) is presented in Figure 4. The operation of the device is based on monitoring the pressure change occurring during melting in an isochoric system, induced by the combined effects of the dissolution of the medium into the solid sample and its melt and the change in the molar volume caused by the phase transition. The pressure difference between the sample holder and the reference cell is detected by a highly sensitive differential pressure transmitter (ARMANO Messtechnik GmbH, Beierfeld, Germany, operational parameters: *T*_max_: 353 K, *p*_max_: 40 MPa, Δ*p*_max_ = 1.6 MPa).

**Figure 4:**
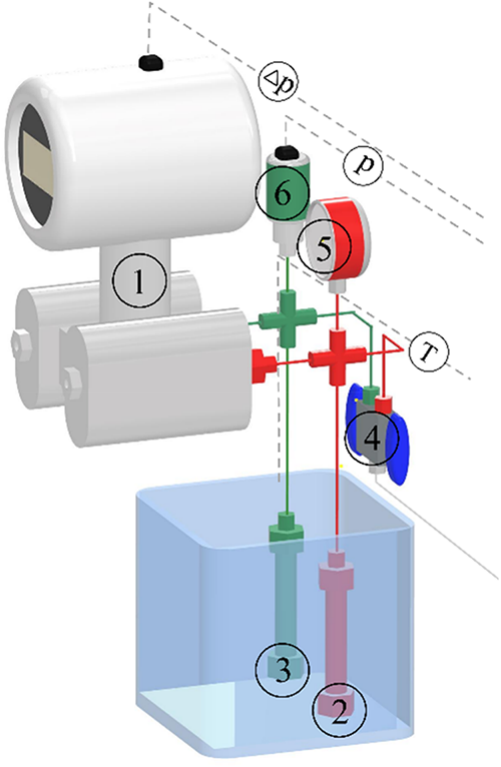
Schematic of the differential pressure method: 1. Differential pressure transmitter, 2. Reference cell, 3. Sample holder cell, 4. Filling valve (CO_2_ pump), 5. Manometer, 6 Pressure transmitter.

At the beginning of the measurement, approximately 500 mg of the solid sample was loaded into a clean sample holder. After assembling the system, both the sample holder and reference cells were pressurized simultaneously using a Teledyne ISCO 260D syringe pump (Teledyne ISCO USA, Nebraska, operational parameters: *T*: 278–318 K, *p*_max_: 51.7 MPa) or with a JASCO PU-1580-CO_2_ pump (ABL&E-JASCO Magyarország, Budapest, Hungary, *p*_max_: 30 MPa). After pressurization, the two cells were isolated from each other and from the CO_2_ supply by closing the valves (4). The pressurized cells (2; 3) were equilibrated in a preheated thermostat (the measurement can only be started when the differential pressure has reached a constant value). Both cells were submerged in the same oil bath, where the temperature was increased by a linear heating profile during each measurement. Temperature was increased by a 10 or 15 K/h heating rate, with the precision Julabo Corio CP-600F thermostat (238 to 473 K, ± 0.03 K). The change in the heating rate did not sharply influence the shape or onset values of the signals. Pressure–temperature–differential pressure data were monitored and logged. The collected data were evaluated by an automated method, also developed in the research group. A detailed description of the operation of the device and the algorithm can be found in a previous publication.^38^ The advantages of the method are its automated operation, the linear heating profile, and the evaluation algorithm similar to that used in differential scanning calorimetry,^47^ which results in a reduction of the error due to human observation.

Among the first steps of the evaluation process, the raw differential pressure data are plotted as a function of temperature after a specially modified moving average smoothing.^47^ The smoothed data are then numerically differentiated with respect to temperature, which results in a peak in the case of the currently investigated model compounds. After linear baseline correction, tangents through the inflection points of the peak are calculated, and the onset and offset values are defined as their intersections with the baseline. The steps of the evaluation can be followed in Figure 5.

**Figure 5:**
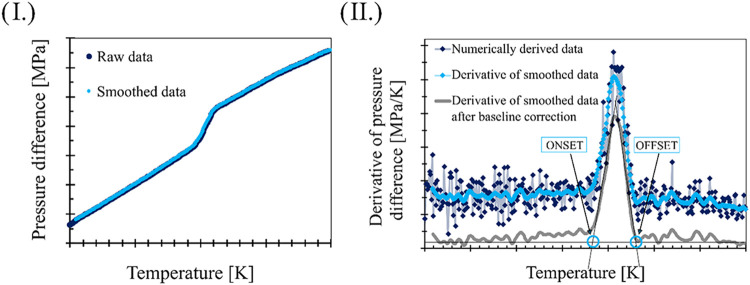
Schematic evaluation of a crystalline compound under carbon dioxide pressure I. Raw and smoothed Δ*p*(*T*)–*T* data, II. Numerically differentiated data.

The standard deviation of the onset values was calculated previously in the case of ibuprofen, benzoic acid,^38^ and, for this study, polycaprolactone. From standard deviation, the expanded measurement uncertainty is calculated according to the *Student’s t*–*distribution* table for α = 0.05. In Table 4, the standard deviations and expanded measurement uncertainties are summarized.

**Table 4:** Measurement Uncertainty of the Differential Pressure Method

	Ibuprofen	Benzoic acid	Polycaprolactone	
Pressure	8 MPa (onset)	16 MPa (onset)	10 MPa (peak max)	10 MPa (offset)
Number of repetitions	6	4	6	6
Standard deviation	0.68 K	0.33 K	1.08 K	1.54 K
Expanded measurement uncertainty	±1.76 K	±0.99 K	±2.18 K	±3.11 K

#### 2.2.4 Ambient Pressure Differential Scanning Calorimetry (DSC)

The atmospheric melting temperatures of the investigated compounds were recorded using a TA Instruments 2920 Modulated DSC (TA Instruments, USA, New Castle) equipment. Roughly 5 mg of the solid sample was filled in the aluminum crucible. The used heating rate was 10 K/min up to 408 K The measurement results served as references to the high-pressure study in order to accurately investigate the melting point depression.

## 3 Results and Discussion

The solid–liquid phase transition of pure compounds was investigated with atmospheric differential scanning calorimetry and under a pressurized carbon dioxide atmosphere with two different high-pressure analytical devices. The pressure- and composition-dependent melting behavior of polycaprolactone–ibuprofen and polycaprolactone–ketoprofen mixtures was measured with the high-pressure view-cell.

### 3.1 Pressure-Dependent Melting Temperature of the Pure Components

#### 3.1.1 Ibuprofen

The melting temperature of ibuprofen was measured in our earlier work.^38^ The aim of that research was to present and validate the automated measurement method that was developed in the research group. Measurements were conducted both using the differential pressure-based apparatus and in the high-pressure view-cell for reference data. The results are shown in Figure 6. Our results fit well with the literature data, and the difference between the temperatures can be caused by the expanded measurement uncertainty.

**Figure 6:**
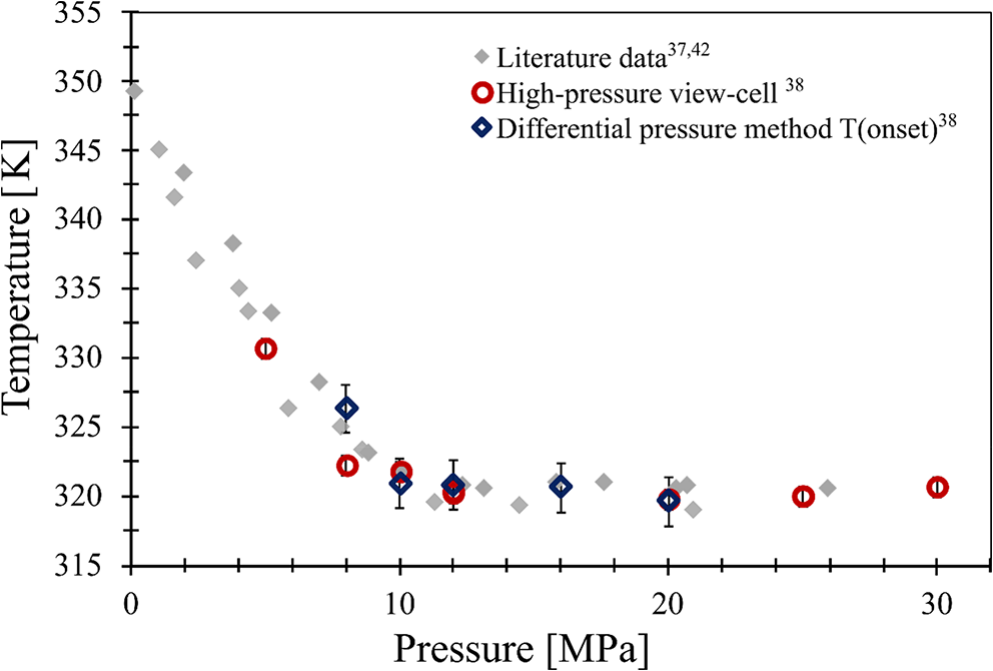
Melting behavior of ibuprofen from literature data (gray diamond)^37,42^ and our previous work: differential pressure method (blue diamond), high-pressure view-cell (red circle). Results can be found in our previous publication.^38^

In the lower pressure range (up to about 10 MPa), the dissolution of CO_2_ dominates, causing a sharp depression in the melting temperature (∼0.1–10 MPa pressure range). At higher pressures, static pressure becomes more dominant, resulting in a pressure-invariant melting temperature (∼10–30 MPa).

#### 3.1.2 Ketoprofen

Ketoprofen, also an NSAID, shows pressure-dependent melting behavior similar to that of ibuprofen. To the best of the authors’ knowledge, the effect of carbon dioxide pressure on the melting behavior of this compound has not been studied earlier. It is observed that the melting temperature of the compound decreases with pressure in the range of 0.1–25 MPa. Figure 7 shows the pressure-dependent melting behavior of ketoprofen. Most of the measurements were taken by the differential pressure method, and only validation experiments were conducted in the view-cell at 3 different pressures (3, 10, and 15 MPa). The detailed results can be found in Supporting Table S8.

**Figure 7:**
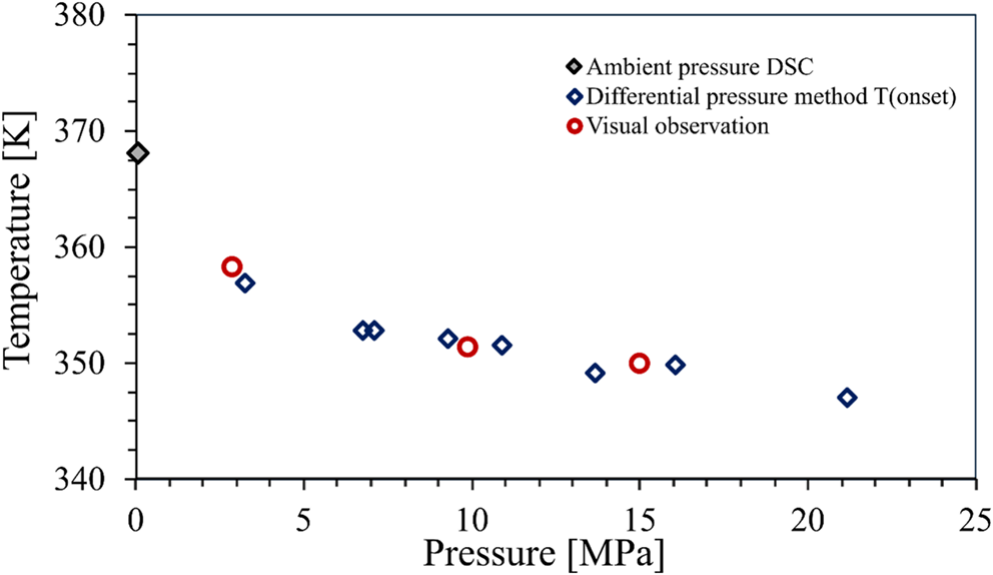
Pressure-dependent melting behavior of ketoprofen under carbon dioxide: differential pressure method (blue diamond), high-pressure view-cell (red circle).

The maximal observed melting point depression (MPD) was approximately 21 K, which is lower than that in the case of ibuprofen. The reason for the lower MPD could be the lower solubility of carbon dioxide in the solid sample.

#### 3.1.3 Polycaprolactone

Besides the investigation of the potential polymer composites, the aim of the current research is also to investigate the applicability of the differential pressure-based measurement device in the case of polymeric samples. The literature data and our results are summarized in Figure 8. The detailed results can be found in Supporting Table S9.

**Figure 8:**
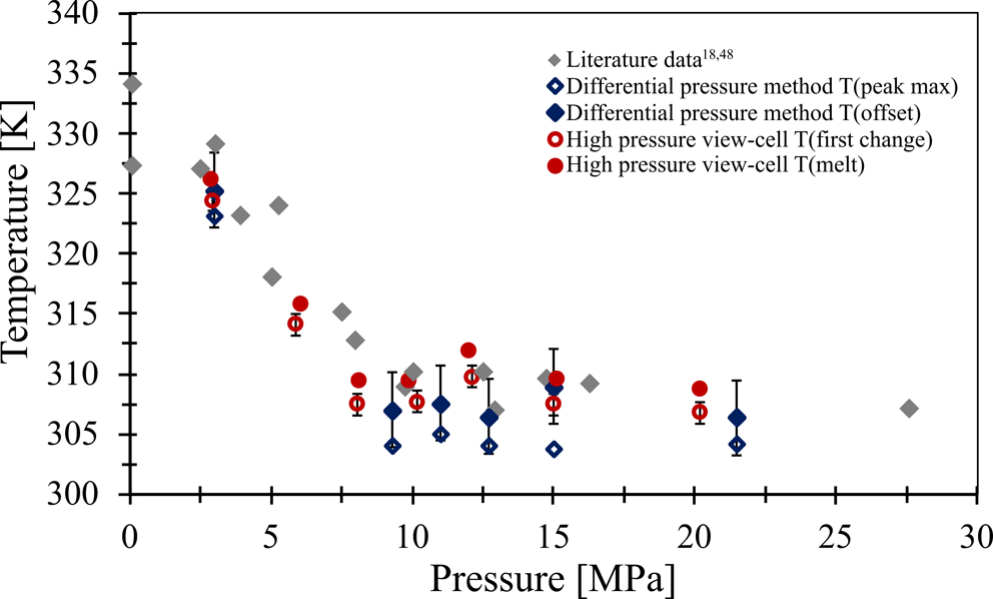
Melting behavior of polycaprolactone under carbon dioxide, literature data (gray diamond),^18,48^ differential pressure method peak maximum (blue empty diamond), offset (blue diamond), high-pressure view-cell first change (empty red circle), melt (red circle).

Melting temperature measured with the high-pressure view-cell shows no significant difference compared to already available literature data obtained by methods such as torsional braid analysis,^18^ visual observation,^48,49^ and even infrared spectroscopy.^50^ But, with the differential-pressure-based method, the onset temperature of the melting curve was significantly lower.

A high-molecular-weight (average molecular weight 80,000 Da, with an unknown molecular size distribution) polycaprolactone sample was chosen for the study. It is a semicrystalline polymer, causing a broad melting range, also making accurate melting point detection challenging. In the case of the polymeric sample, the extrema and offset value of the melting peak were used as a basis of evaluation. Although the intervals defined by the expanded measurement uncertainty of both methods overlap, the temperature readings of the differential-pressure-based method seem consistently lower than the last melting points observed in the view-cell. An underlying phenomenon is implied. The thermomechanical curve of polymers is shown in Figure 9 based on the publication of Pei et al.^51^

**Figure 9:**
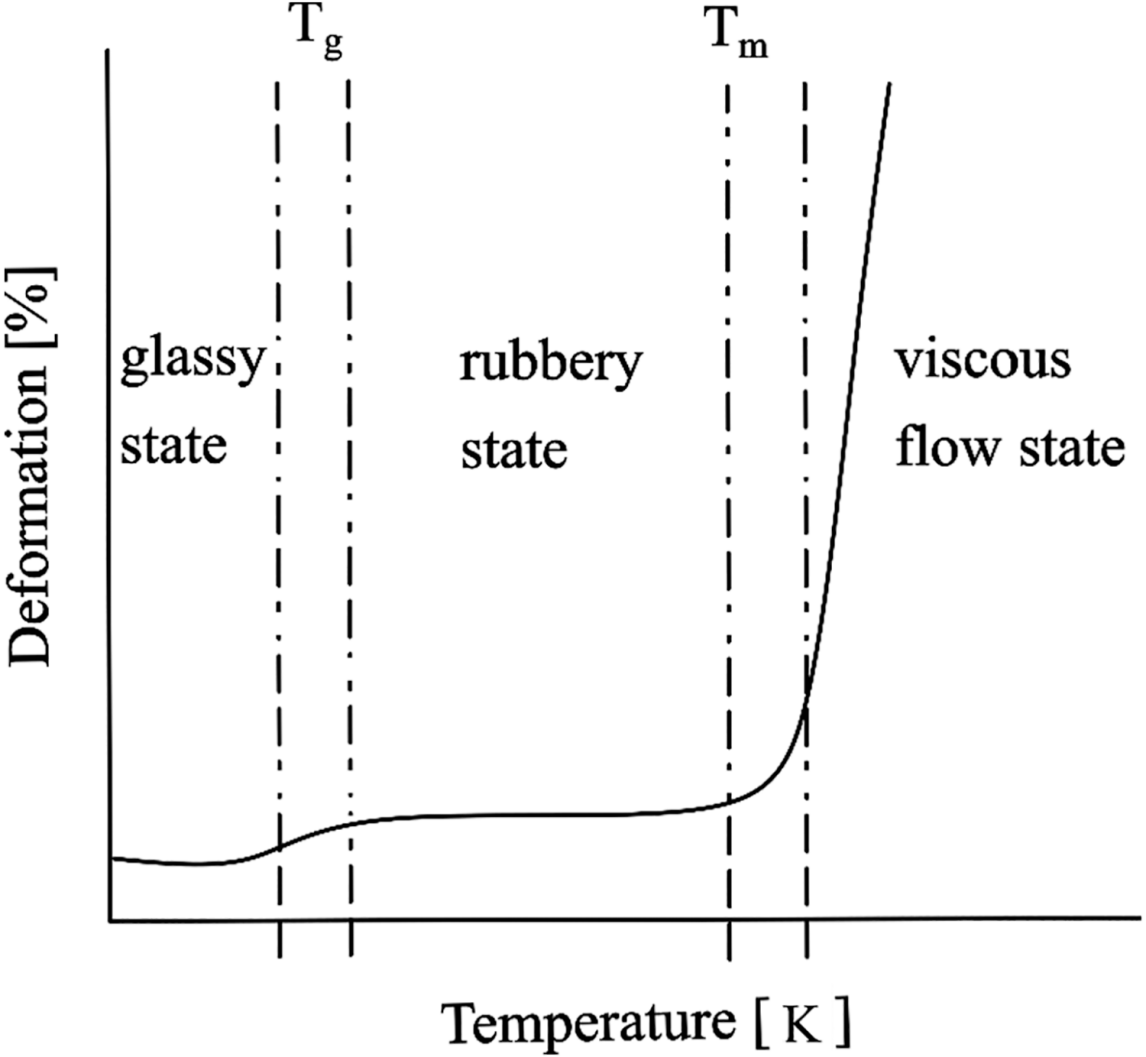
Thermomechanical curve of polymers^51^

The signal caused by the dissolution of carbon dioxide into the polymer sample is assumed to correspond to the melting temperature range of the polymer (*i.e*., the temperature range marked by *T*_m_ in Figure 9). This occurs at lower temperatures than the visibly observed radical change (*i.e*., the sample turning translucent and then losing its defined shape). Thus, while the differential pressure-based method may be able to detect the melting temperature interval, the data provided by view-cell measurements show that melting temperatures must be overestimated for polymer processing. The stages of melting of a polymeric sample are shown in Figure 10.

**Figure 10:**
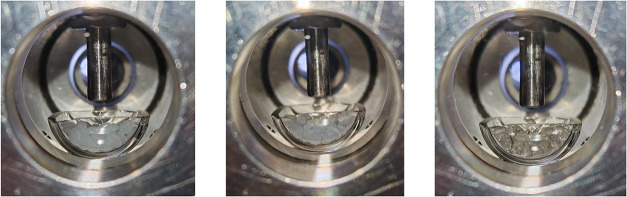
Stages of the melting of polycaprolactone under 10 MPa carbon dioxide: (Left) Beginning of the measurement (rubbery state), (middle) beginning of the rubbery–viscous flow state transition, and (right) detected melting point (supposed end of the rubbery–viscous flow state transition).

### 3.2 Pressure- and Composition-Dependent Melting Behavior of API–PCL Systems

Many systems retain their eutectic behavior in a high-pressure carbon dioxide atmosphere. Often, a decrease in the melting point and eutectic temperature can be observed, but the eutectic composition is practically independent of pressure. Figure 11 shows a schematic diagram illustrating the number of phases in a eutectic system containing two solid components and supercritical carbon dioxide as functions of pressure and composition. The composition axis represents the mass fraction of component *A* of the solid, not taking into account dissolved carbon dioxide.

**Figure 11:**
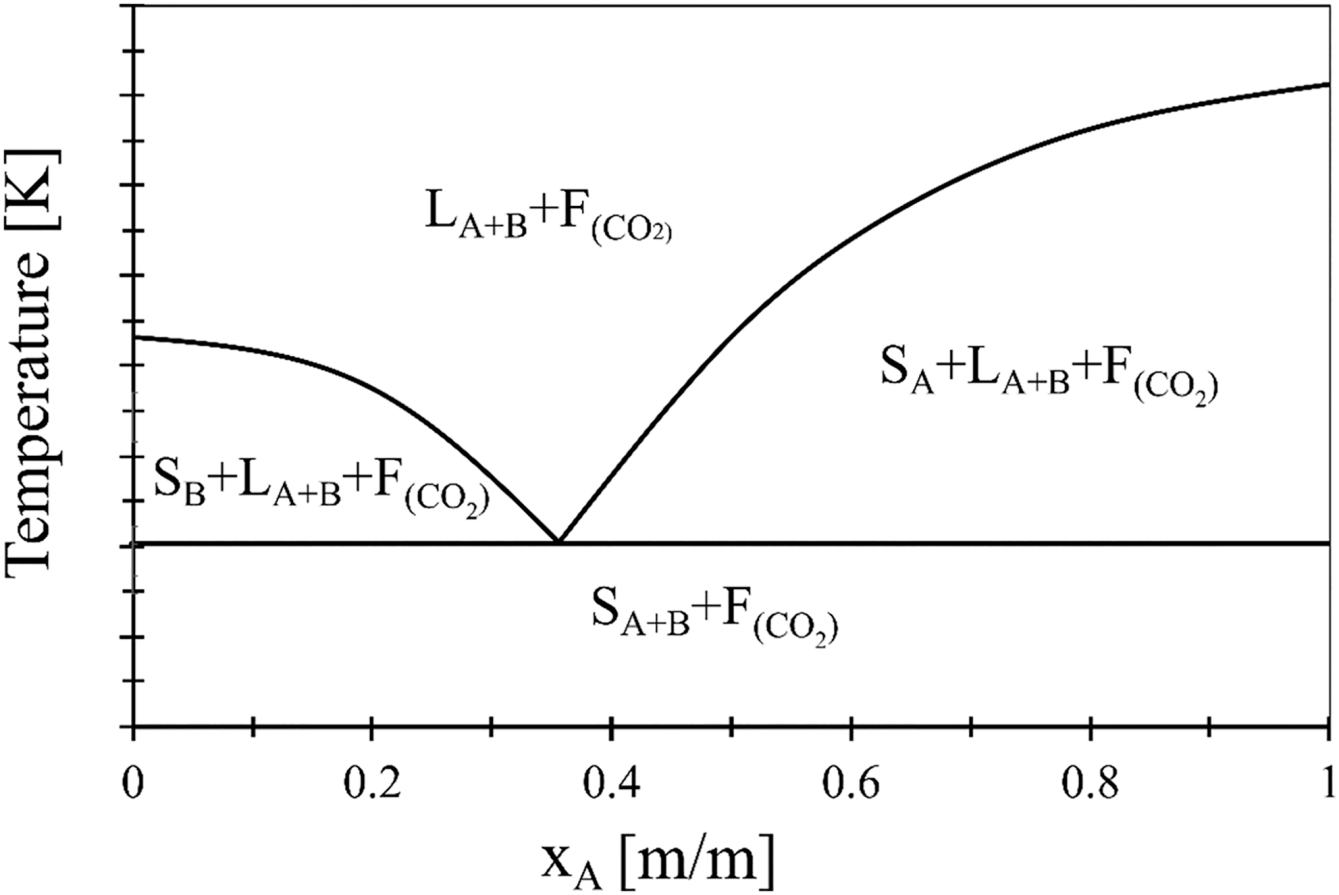
Schematic diagram illustrating the number of phases in a system containing two solid components and supercritical carbon dioxide as a function of pressure and composition.

Both the ibuprofen–polycaprolactone and ketoprofen–polycaprolactone systems were investigated at three different pressures (3, 10, and 15 MPa). The first measurements on the composition-dependent phase diagrams were conducted at 10 MPa as the melting behavior of the pure components suggests that the effect of pressure is not significant beyond this pressure value. Melting behavior was also investigated at 15 MPa to ensure that the maximal melting point depression was reached. The effect of a gaseous CO_2_ atmosphere was studied at 3 MPa.

#### 3.2.1 Ibuprofen-PCL

No existing experiment series has been found for the composition-dependent melting behavior of the ibuprofen polycaprolactone system over the full composition range at atmospheric pressure. But, it has been observed in some articles on the development of controlled-release systems that the melting point of systems containing both compounds is lower than that of any of the pure components. In DSC curves found in some different literature sources, for different ibuprofen to PCL ratios, two peaks appear, suggesting eutectic behavior.^52–54^

The pressure- and composition-dependent results of the high-pressure experiment series are shown in Figure 12. Measurements have been conducted in the high-pressure view-cell, and the data from the experiments can be found in Tables S2–S4.

**Figure 12:**
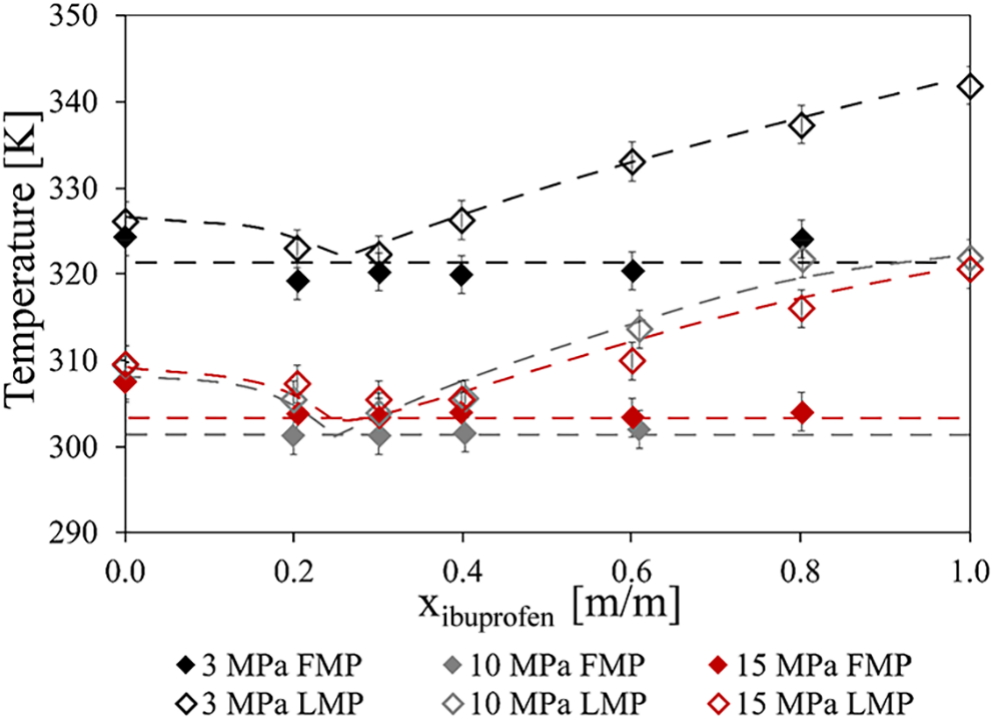
Pressure- and composition-dependent results of the ibuprofen-PCL system: empty diamonds mark the first melting point (FMP): black: 3 MPa system pressure, gray: 10 MPa, red: 15 MPa. Solid diamonds show the last melting point (LMP): black 3 MPa, gray 10 MPa, red 15 MPa. The dashed lines in the figure do not show computational or modeling results; they only serve the ease of reading.

Two distinct melting phenomena could be observed, one plotted as the FMP values and the other shown as the LMP. The FMP values appearing constant regardless of sample composition suggests eutectic behavior, albeit no change was seen at the assumed eutectic temperature at 3 and 10 MPa for the sample with 80 m/m% ibuprofen. The melting and eutectic temperatures show a pressure dependence similar to that of the pure compounds: significantly higher values were measured in lower-pressure media (3 MPa) than in the presence of 10 and 15 MPa carbon dioxide. In the case of ibuprofen as the API, it appears that the melting temperatures of the system measured at 10 MPa are significantly lower than the results at 3 MPa. The results of the measurements at 15 MPa appear to be slightly higher than those at 10 MPa, but such a slight difference may not be significant as the expanded measurement uncertainty is ±2.21 K based on repeated measurements of pure polycaprolactone. The assumed eutectic composition occurs mostly at the same composition, approximately 30 m/m% ibuprofen. The dashed lines in the figure do not show computational or modeling results; they only serve the ease of reading. Results show that 10 MPa can be an appropriate parameter for PGSS, and at this pressure, a maximum of MPD is reached at any composition. Hence, further pressure increase does not aid in the avoidance of thermal degradation; it could affect the particle sizes of the product.

#### 3.2.2 Ketoprofen-PCL

The pressure- and composition-dependent melting phase diagram of the ketoprofen–polycaprolactone system is shown in Figure 13, with the dashed solidus and liquidus lines drawn as a guide to the eye. The data from the experiments can be found in Tables S5–S7.

**Figure 13:**
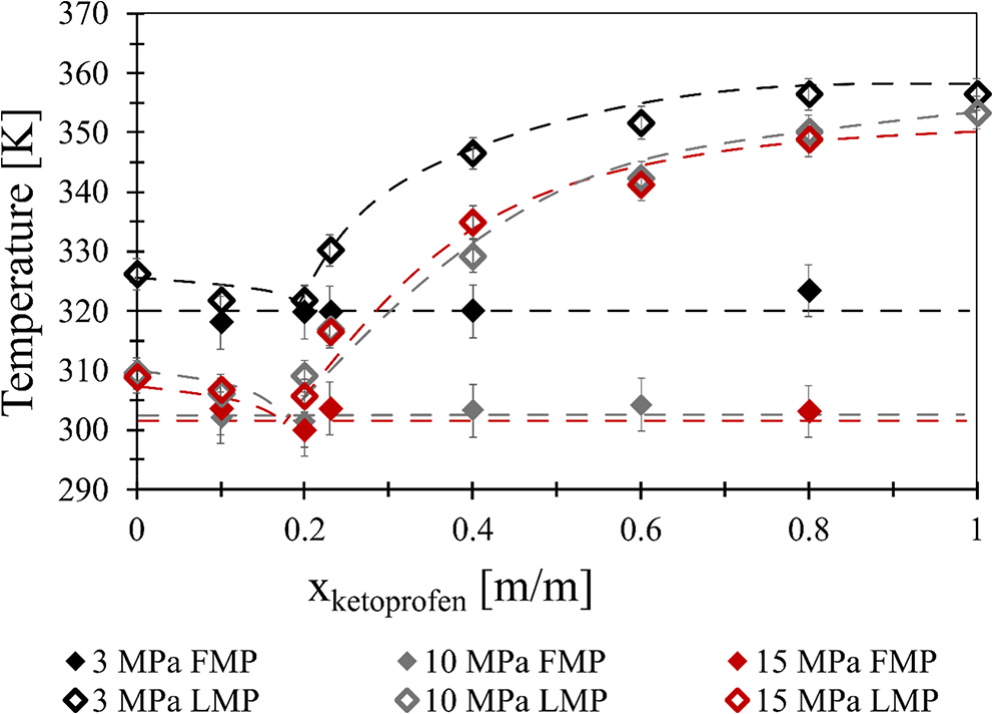
Pressure- and composition-dependent melting phase diagram of ketoprofen–polycaprolactone. Empty diamonds show the first melting point (FMP): black 3 MPa, system pressure, gray 10 MPa, red 15 MPa. Solid diamonds mark the last melting point (LMP): black 3 MPa, gray 10 MPa, red 15 MPa. The dashed lines in the figure do not show computational or modeling results; they only serve the ease of reading.

Similar observations can be made for the ibuprofen-containing mixture, although the FMP associated with the eutectic temperature was much less pronounced. The assumed eutectic temperature decreases with increasing pressure as well as the melting temperatures. The assumed eutectic composition does not change significantly. A much higher melting temperature can be observed in the 3 MPa measurements, but the difference between the 10 and 15 MPa measurements is not significant, considering the expanded measurement uncertainty. 10 MPa might be feasible for the operating pressure of the micronization process of a ketoprofen–PCL composite. From a solely technological point of view, if a molten sample is needed for crystallization or production of a controlled particle size product, working with the composition with the lowest melting point is the most energy efficient. In the case of the ketoprofen-polycaprolactone system, this composition is around 25 m/m% of ketoprofen.

### 3.3 Atmospheric DSC Measurements

Atmospheric DSC measurements were conducted to confirm the eutectic behavior of the systems and to provide the reference data to accurately evaluate melting point depression in the high-pressure media. Although the DSC curves do not show complete eutectic behavior with absolute certainty, the first melting point of the composites is at a temperature lower than that of the pure components. The DSC curves of the different composites are shown in Figure 14.

**Figure 14:**
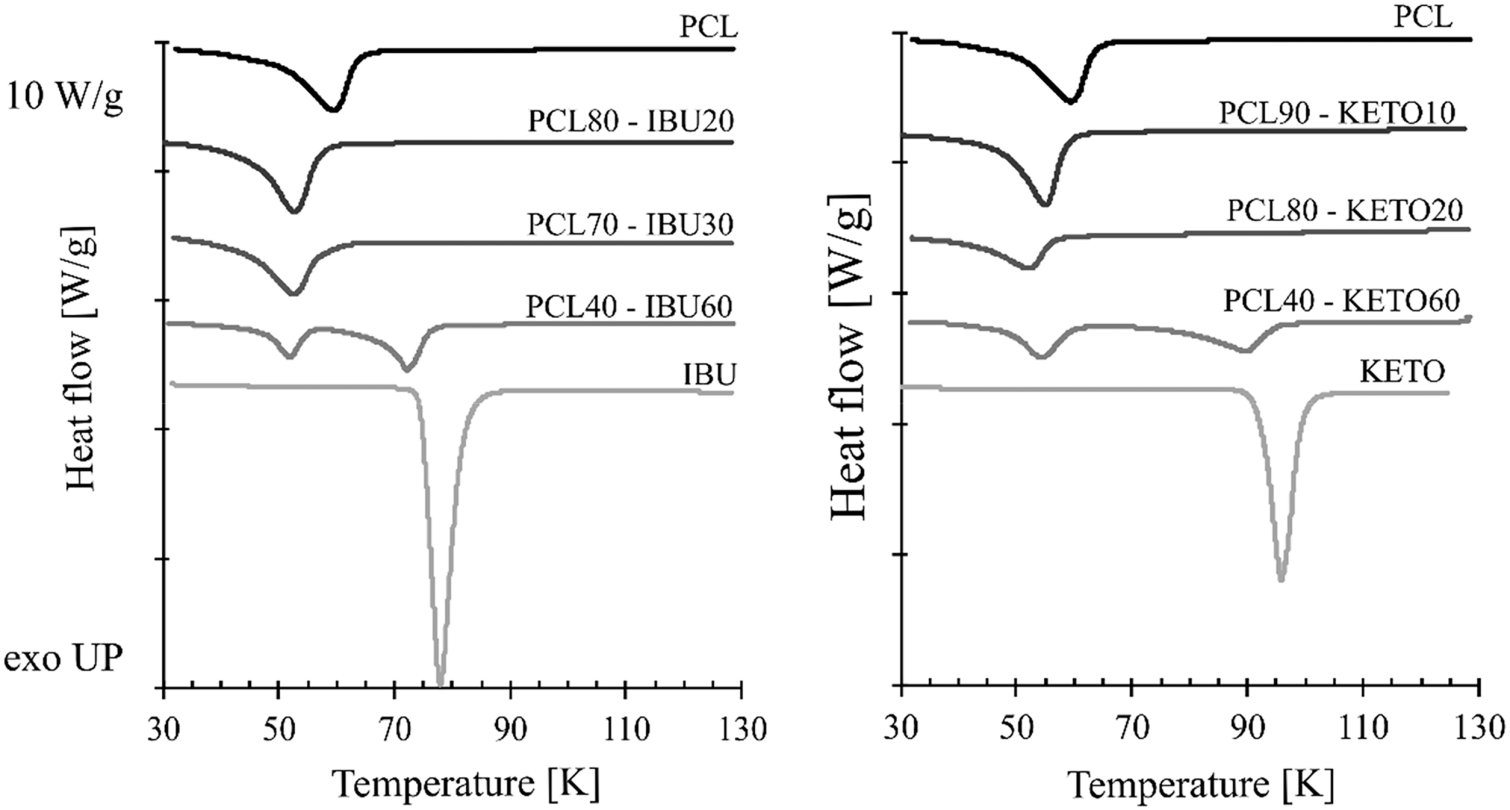
DSC curves of different polycaprolactone-API composites.

Two peaks, typical for a eutectic system, were observed for composites, which are richer in API than the the assumed eutectic composition. Although only one peak is observed in the lower active ingredient compositions, this does not completely contradict the eutectic behavior. It may be possible that the two peaks completely overlap, even if the sample melts within a 4–6 K temperature range. The detailed results can be found in Supporting Tables S10–S11.

## 4 Conclusion

Thermodynamic data were measured, which may be key to the design of controlled drug delivery systems. Melting behavior measurements were performed in high-pressure carbon dioxide at 3, 10, and 15 MPa to support the design of a possible PGSS process. The pressure-dependent melting behavior of pure ketoprofen and polycaprolactone was studied using a differential pressure measurement method developed in the research group, and with a high-pressure view-cell, the results on ibuprofen were obtained earlier. In CO_2_ atmosphere, each component showed sharp melting point depression up to 10 MPa.

The pressure- and composition-dependent melting behavior of ibuprofen–polycaprolactone and ketoprofen–polycaprolactone systems was also investigated. The melting point decreasing effect of carbon dioxide was demonstrated on the composition-dependent phase diagrams, where eutectic behavior has been observed. Increasing the pressure did not significantly affect the eutectic composition.

## Supplementary Material



## Notes

Results of the melting temperature measurements; data on the pressure-dependent melting temperatures of ketoprofen, polycaprolactone, and the pressure- and composition-dependent melting temperatures of the polymer-NSAID mixtures; and uncertainty of the equipment used in the experimental determination of the data (PDF)
