# Supplementary material for: Phase Behavior of Potential Drug Delivery Systems, Polycaprolactone—Nonsteroidal Anti-Inflammatory Drug in a Pressurized Carbon Dioxide Medium
Source: ACS Omega. 2025 Dec 17;10(51):62657–66. doi: 10.1021/acsomega.5c06958 (PMC12756719; doi:10.1021/acsomega.5c06958)
Supplement: Supplementary file 1 [file ao5c06958_si_001.pdf]

Supplementary data for the manuscript:

„Phase behavior of potential drug delivery systems, polycaprolactone – Non-Steroidal Anti-Inflammatory Drug in a pressurized carbon dioxide medium”

The measurement uncertainties of the pressure transmitter, thermometer, differential pressure transmitter, analytical balance and differential scanning calorimeter can be found in the following table.

Table S1. *Measurement uncertainties*

Used device	Accuracy/Uncertainty (determined by the manufacturer)
K-type thermometer	≤ 0.3 % (maximum ± 1 K)
Fitted calibration line	$T_{calc} = 0.9954 \cdot T_{cell} + 1.2104$ [K]
Pressure transmitter	≤ 0.5 % of span limit point calibration (0.25 % of span best fit straight line)
Differential pressure transmitter	≤0.075 %
Analytical balance	± 0.1 mg
Differential Scanning Calorimetry	± 0.1 K

Table S2. *Solid-liquid phase transition of the PCL-ibuprofen system under ~10 MPa CO_2 pressure*

$w_{ibuprofen}$	p_1 [MPa]	T_1[K]	phase transition	p_2 [MPa]	T_2[K]	phase transition
0.000	10.2	307.65	$s+f_{(CO2)} \rightarrow s+l+f_{(CO2)}$	9.9	309.45	$s+l+f_{(CO2)} \rightarrow l+f_{(CO2)}$
0.199	10.3	301.25	$s+f_{(CO2)} \rightarrow s+l+f_{(CO2)}$	10.2	305.45	$s+l+f_{(CO2)} \rightarrow l+f_{(CO2)}$
0.301	9.9	301.35	$s+f_{(CO2)} \rightarrow s+l+f_{(CO2)}$	10.3	303.95	$s+l+f_{(CO2)} \rightarrow l+f_{(CO2)}$
0.403	9.91	301.55	$s+f_{(CO2)} \rightarrow s+l+f_{(CO2)}$	9.97	305.55	$s+l+f_{(CO2)} \rightarrow l+f_{(CO2)}$
0.609	9.97	301.95	$s+f_{(CO2)} \rightarrow s+l+f_{(CO2)}$	10	313.65	$s+l+f_{(CO2)} \rightarrow l+f_{(CO2)}$
0.803	m.d.	m.d.	m.d.	10.1	321.75	$s+l+f_{(CO2)} \rightarrow l+f_{(CO2)}$
1.000	-	-	-	10.1	321.85 [1]	$s+f_{(CO2)} \rightarrow l+f_{(CO2)}$

Table S3. *Solid-liquid phase transition of the PCL-ibuprofen system under ~15 MPa CO_2 pressure*

$w_{ibuprofen}$	p_1 [Mpa]	T_1[K]	phase transition	p_2 [Mpa]	T_2[K]	phase transition
0.000	15.0	307.45	$s+f_{(CO2)} \rightarrow s+l+f_{(CO2)}$	15.0	309.55	$s+l+f_{(CO2)} \rightarrow l+f_{(CO2)}$
0.205	14.9	303.85	$s+f_{(CO2)} \rightarrow s+l+f_{(CO2)}$	15.3	307.25	$s+l+f_{(CO2)} \rightarrow l+f_{(CO2)}$
0.301	15.3	303.35	$s+f_{(CO2)} \rightarrow s+l+f_{(CO2)}$	15.0	305.45	$s+l+f_{(CO2)} \rightarrow l+f_{(CO2)}$
0.398	14.8	304.05	$s+f_{(CO2)} \rightarrow s+l+f_{(CO2)}$	15.2	305.45	$s+l+f_{(CO2)} \rightarrow l+f_{(CO2)}$
0.601	14.9	303.35	$s+f_{(CO2)} \rightarrow s+l+f_{(CO2)}$	14.9	309.95	$s+l+f_{(CO2)} \rightarrow l+f_{(CO2)}$
0.802	15.3	304.05	$s+f_{(CO2)} \rightarrow s+l+f_{(CO2)}$	14.8	316.05	$s+l+f_{(CO2)} \rightarrow l+f_{(CO2)}$
1.000	-	-	-	15.0	320.55 [1]	$s+f_{(CO2)} \rightarrow l+f_{(CO2)}$

Table S4. *Solid-liquid phase transition of the PCL-ibuprofen system under ~3 MPa CO_2 pressure*

$w_{ibuprofen}$	p_1 [Mpa]	T_1[K]	phase transition	p_2 [Mpa]	T_2[K]	phase transition
0.000	2.96	324.35	$s+f_{(CO2)} \rightarrow s+l+f_{(CO2)}$	2.88	326.15	$s+l+f_{(CO2)} \rightarrow l+f_{(CO2)}$
0.205	3.01	319.95	$s+f_{(CO2)} \rightarrow s+l+f_{(CO2)}$	2.86	322.95	$s+l+f_{(CO2)} \rightarrow l+f_{(CO2)}$
0.205	3.05	319.25	$s+f_{(CO2)} \rightarrow s+l+f_{(CO2)}$	3.05	322.25	$s+l+f_{(CO2)} \rightarrow l+f_{(CO2)}$
0.205	2.99	318.95	$s+f_{(CO2)} \rightarrow s+l+f_{(CO2)}$	2.99	332.25	$s+l+f_{(CO2)} \rightarrow l+f_{(CO2)}$
0.301	3.10	320.25	$s+f_{(CO2)} \rightarrow s+l+f_{(CO2)}$	3.10	322.25	$s+l+f_{(CO2)} \rightarrow l+f_{(CO2)}$
0.398	2.95	319.95	$s+f_{(CO2)} \rightarrow s+l+f_{(CO2)}$	2.93	326.25	$s+l+f_{(CO2)} \rightarrow l+f_{(CO2)}$
0.601	2.95	320.35	$s+f_{(CO2)} \rightarrow s+l+f_{(CO2)}$	2.91	333.05	$s+l+f_{(CO2)} \rightarrow l+f_{(CO2)}$
0.802	3.11	324.05	$s+f_{(CO2)} \rightarrow s+l+f_{(CO2)}$	3.10	337.35	$s+l+f_{(CO2)} \rightarrow l+f_{(CO2)}$
1.000	-	-	-	3.13	341.85	$s+f_{(CO2)} \rightarrow l+f_{(CO2}$

Table S5. *Solid-liquid phase transition of the PCL-ketoprofen system under ~10 MPa CO_2 pressure*

$w_{ketoprofen}$	p_1 [Mpa]	T_1[K]	phase transition	p_2 [Mpa]	T_2[K]	phase transition
0.000	10.2	307.65	s+f$_{(CO2)}$ → s+l+f$_{(CO2)}$	9.90	309.45	s+l+f$_{(CO2)}$ → l+f$_{(CO2)}$
0.102	10.3	302.05	s+f$_{(CO2)}$ → s+l+f$_{(CO2)}$	10.1	306.05	s+l+f$_{(CO2)}$ → l+f$_{(CO2)}$
0.201	10.21	299.35	s+f$_{(CO2)}$ → s+l+f$_{(CO2)}$	10.2	309.05	s+l+f$_{(CO2)}$ → l+f$_{(CO2)}$
0.201	9.88	301.95	s+f$_{(CO2)}$ → s+l+f$_{(CO2)}$	9.74	307.85	s+l+f$_{(CO2)}$ → l+f$_{(CO2)}$
0.201	10.1	302.35	s+f$_{(CO2)}$ → s+l+f$_{(CO2)}$	10.2	308.15	s+l+f$_{(CO2)}$ → l+f$_{(CO2)}$
0.201	9.93	301.85	s+f$_{(CO2)}$ → s+l+f$_{(CO2}$	m.d.	m.d.	m.d.
0.240	10.1	303.65	s+f$_{(CO2)}$ → s+l+f$_{(CO2)}$	10.3	316.85	s+l+f$_{(CO2)}$ → l+f$_{(CO2)}$
0.397	10.2	303.25	s+f$_{(CO2)}$ → s+l+f$_{(CO2)}$	10.4	329.25	s+l+f$_{(CO2)}$ → l+f$_{(CO2)}$
0.599	10.2	304.25	s+f$_{(CO2)}$ → s+l+f$_{(CO2)}$	10.1	342.35	s+l+f$_{(CO2)}$ → l+f$_{(CO2)}$
0.799	m.d.	m.d.	m.d.	10.1	350.15	s+l+f$_{(CO2)}$ → l+f$_{(CO2)}$
1.000	-	-	-	9.9	351.45	s+f$_{(CO2)}$ → l+f$_{(CO2}$

Table S6. *Solid-liquid phase transition of the PCL-ketoprofen system under ~15 MPa CO_2 pressure*

$w_{ketoprofen}$	p_1 [Mpa]	T_1[K]	phase transition	p_2 [Mpa]	T_2[K]	phase transition
0.000	15.0	303.65	s+f$_{(CO2)}$ → s+l+f$_{(CO2)}$	15.0	308.90	s+l+f$_{(CO2)}$ → l+f$_{(CO2)}$
0.102	15.1	303.65	s+f$_{(CO2)}$ → s+l+f$_{(CO2)}$	15.1	306.65	s+l+f$_{(CO2)}$ → l+f$_{(CO2)}$
0.201	15.0	299.95	s+f$_{(CO2)}$ → s+l+f$_{(CO2)}$	15.3	305.75	s+l+f$_{(CO2)}$ → l+f$_{(CO2)}$
0.240	15.2	303.65	s+f$_{(CO2)}$ → s+l+f$_{(CO2)}$	15.2	316.55	s+l+f$_{(CO2)}$ → l+f$_{(CO2)}$
0.397	m.d.	m.d.	m.d.	15.2	334.95	s+l+f$_{(CO2)}$ → l+f$_{(CO2)}$
0.599	m.d.	m.d.	m.d.	15.2	342.25	s+l+f$_{(CO2)}$ → l+f$_{(CO2)}$
0.799	14.9	303.15	s+f$_{(CO2)}$ → s+l+f$_{(CO2)}$	15.0	348.75	s+l+f$_{(CO2)}$ → l+f$_{(CO2)}$
1.000	-	-	-	15.0	350.05	s+l+f$_{(CO2)}$ → l+f$_{(CO2)}$

Table S7. *Solid-liquid phase transition of the PCL-ketoprofen system under ~3 MPa CO_2 pressure*

$w_{ketoprofen}$	p_1 [Mpa]	T_1[K]	phase transition	p_2 [Mpa]	T_2[K]	phase transition
0.000	2.96	324.5	s+f$_{(CO2)}$ → s+l+f$_{(CO2)}$	2.88	326.15	s+l+f$_{(CO2)}$ → l+f$_{(CO2)}$
0.102	2.79	318.05	s+f$_{(CO2)}$ → s+l+f$_{(CO2)}$	2.92	321.75	s+l+f$_{(CO2)}$ → l+f$_{(CO2)}$
0.201	3.16	319.75	s+f$_{(CO2)}$ → s+l+f$_{(CO2)}$	3.00	321.75	s+l+f$_{(CO2)}$ → l+f$_{(CO2)}$
0.240	3.04	319.75	s+f$_{(CO2)}$ → s+l+f$_{(CO2)}$	3.09	330.25	s+l+f$_{(CO2)}$ → l+f$_{(CO2)}$
0.397	3.06	319.65	s+f$_{(CO2)}$ → s+l+f$_{(CO2)}$	3.13	346.55	s+l+f$_{(CO2)}$ → l+f$_{(CO2)}$
0.599	m.d.	m.d.	m.d.	3.10	351.65	s+l+f$_{(CO2)}$ → l+f$_{(CO2)}$
0.799	3.2	323.45	s+f$_{(CO2)}$ → s+l+f$_{(CO2)}$	3.06	356.45	s+l+f$_{(CO2)}$ → l+f$_{(CO2)}$
1.000	-	-	-	2.99	356.35	s+l+f$_{(CO2)}$ → l+f$_{(CO2)}$

Table S8. *Solid-liquid phase transition of ketoprofen under CO_2 pressure*

P [MPa]	$T_{melting}$ [K]	*method*
3.30	356.95	differential pressure method
6.80	352.75	differential pressure method
7.10	352.85	differential pressure method
9.30	352.05	differential pressure method
10.9	351.55	differential pressure method
13.7	349.15	differential pressure method
16.10	349.85	differential pressure method
21.2	346.95	differential pressure method
9.90	351.45	view-cell
15.0	350.05	view-cell
2.88	358.35	view-cell

Table S9. *Solid-liquid phase transition of PCL under CO_2 pressure*

p [MPa]	T_{onset}*[K]*	*p [MPa]*	$T_{peak\ max}$*[K]*	*p [MPa]*	T_{offset}*[K]*	*method*
3.00	319.4	2.98	323.1	3.03	325.24	differential pressure method
9.30	300.9	9.5	304	9.80	307.0	differential pressure method
11.0	301.63	11.3	305	11.4	307.58	differential pressure method
12.7	302.88	12.8	304	12.9	306.47	differential pressure method
15.0	301.35	15	303.8	15.0	308.93	differential pressure method
21.5	302.01	21.6	304.2	21.7	306.4	differential pressure method

p [MPa]	T_{FMP}*[K]*	*p [MPa]*	T_{LMP}*[K]*	*method*
2.96	324.4	2.88	326.2	view-cell
5.90	314.1	6.03	315.8	view-cell
8.04	307.5	8.10	309.4	view-cell
10.2	307.7	9.89	309.5	view-cell
12.1	309.8	12.0	311.9	view-cell
15.0	307.5	15.1	309.6	view-cell
20.2	306.8	20.2	308.7	view-cell
0.10	334.15	m.d.	m.d.	literature data [2]
2.50	327.15	m.d.	m.d.	literature data [2]

p [MPa]	T_{FMP}*[K]*	*p [MPa]*	T_{LMP}*[K]*	*method*
7.50	315.15	m.d.	m.d.	literature data [2]
10.0	310.15	m.d.	m.d.	literature data [2]
12.5	310.15	m.d.	m.d.	literature data [2]
0.1	327.4	m.d.	m.d.	literature data [3]
3.00	329.1	m.d.	m.d.	literature data [3]
3.88	323.2	m.d.	m.d.	literature data [3]
5.23	324.0	m.d.	m.d.	literature data [3]
7.98	312.8	m.d.	m.d.	literature data [3]
9.73	309.0	m.d.	m.d.	literature data [3]
12.93	307.0	m.d.	m.d.	literature data [3]
14.73	309.6	m.d.	m.d.	literature data [3]
16.27	309.2	m.d.	m.d.	literature data [3]
27.6	307.2	m.d.	m.d.	literature data [3]

Table S10. *DSC measurement data of PCL-ibuprofen system under ambient pressure*

$w_{ibuprofen}$	*peak1*		*peak2*	
	$T_{onset}[K]$	$T_{peak\ max}[K]$	$T_{onset}[K]$	$T_{peak\ max}[K]$
1.00	347.87	351.06	-	-
0.61	320.68	325.04	341.17	345.38
0.30	317.66	325.68	-	-
0.20	318.33	325.8	-	-
0.00	322.41	332.56	-	-

Table S11. *DSC measurement data of PCL-ketoprofen system under ambient pressure*

$w_{ketoprofen}$	*peak1*		*peak2*	
	$T_{onset}[K]$	$T_{peak\ max}[K]$	$T_{onset}[K]$	$T_{peak\ max}[K]$
1.00	365.5	369	-	-
0.60	321.41	327.5	351.42	362.74
0.20	315.42	325.68	-	-
0.10	320.7	328.25	-	-
0.00	322.41	332.56	-	-

[1] D. Arany, M. Kőrösi, E. Székely. A new, automated method for the investigation of melting point depression under carbon dioxide pressure. *Journal of CO_2 Utilization*, (2024) 80, 102663. https://doi.org/10.1016/j.jcou.2023.102663.

[2] D. D. Rhee, E. M. Troiano, G. Floyd. E. Kiran. Melting and crystallization temperatures. foaming. and fluid-induced crystallization of poly (ε-caprolactone) in compressed CO_2 and N_2. *J Supercrit Fluids* 211 (2024) 106293. https://doi.org/10.1016/j.supflu.2024.106293.

[3] E. De Paz, Á. Martín, S. Rodríguez-Rojo, J. Herreras, M.J. Cocero. Determination of phase equilibrium (solid-liquid-gas) in poly-(ε-caprolactone)-carbon dioxide systems. *J Chem Eng Data* 55 (2010) 2781–2785. https://doi.org/10.1021/je900997t.
